# Supplementary material for: A Gold Standard, CRISPR/Cas9-Based Complementation Strategy Reliant on 24 Nucleotide Bookmark Sequences
Source: Genes (Basel). 2020 Apr 23;11(4):458. doi: 10.3390/genes11040458 (PMC7230483; doi:10.3390/genes11040458)
Supplement: Supplementary file 1 [file genes-11-00458-s001.zip › Supplementary.docx]

# Supporting information

## Primer design and gene synthesis

The following primers were used in this study:

**Table S1**. Primers summary.

| Primer | Sequence | Usage |
| --- | --- | --- |
| oFS39 | atcttaaggaggagttttcgtcgacAGGGTTGTGGGTTGTACGGAgttttagagctagaaatagcaagtt | Anneal with oFS79 to assemble BM4 dsDNA oligomer. |
| oFS40 | atcttaaggaggagttttcgtcgacATTTCTGATATTACTGTCACgttttagagctagaaatagcaagtt | Anneal with oFS80 to assemble BM5 dsDNA oligomer. |
| oFS41 | atcttaaggaggagttttcgtcgacACCGATACCGTTTACGAAATgttttagagctagaaatagcaagtt | Anneal with oFS81 to assemble BM6 dsDNA oligomer. |
| oFS42 | atcttaaggaggagttttcgtcgacTGAAGATCAGGCTATCACTGgttttagagctagaaatagcaagtt | Anneal with oFS82 to assemble BM7 dsDNA oligomer. |
| oFS43 | atcttaaggaggagttttcgtcgacTCCGGAGCTCCGATAAAAAAgttttagagctagaaatagcaagtt | Anneal with oFS83 to assemble BM8 dsDNA oligomer. |
| oFS44 | atcttaaggaggagttttcgtcgacTATTGATTCTCTTCAAGTAGgttttagagctagaaatagcaagtt | Anneal with oFS84 to assemble BM9 dsDNA oligomer. |
| oFS45 | atcttaaggaggagttttcgtcgacCCATTGTACTATCATGCTAGgttttagagctagaaatagcaagtt | Anneal with oFS85 to assemble BM10 dsDNA oligomer. |
| oFS46 | atcttaaggaggagttttcgtcgacATGCAGTCGGCTGTAGAAAGgttttagagctagaaatagcaagtt | Anneal with oFS86 to assemble BM11 dsDNA oligomer. |
| oFS47 | atcttaaggaggagttttcgtcgacCGACTGCATTTTATTATGTAgttttagagctagaaatagcaagtt | Anneal with oFS87 to assemble BM12 dsDNA oligomer. |
| oFS66 | atacataaatgcgatcgcGTACCGCTGCTATCTGCC | Amplify *pyrE* homology-directed recombination cassette with oFS67. |
| oFS67 | atacataaatgcgatcgcGTACCGCTGCTATCTGCC | Amplify *pyrE* homology-directed recombination cassette with oFS66. |
| oFS73 | CTAGATTTATATTTAGTCCCTTGCCTTGC | Sequencing of sgRNA cassette. |
| oFS79 | aacttgctatttctagctctaaaacTACATAATAAAATGCAGTCGGTCGACgaaaactcctccttaagat | Anneal with oFS39 to assemble BM4 dsDNA oligomer. |
| oFS80 | aacttgctatttctagctctaaaacCTTTCTACAGCCGACTGCATGTCGACgaaaactcctccttaagat | Anneal with oFS40 to assemble BM5 dsDNA oligomer. |
| oFS81 | aacttgctatttctagctctaaaacCTAGCATGATAGTACAATGGGTCGACgaaaactcctccttaagat | Anneal with oFS41 to assemble BM6 dsDNA oligomer. |
| oFS82 | aacttgctatttctagctctaaaacCTACTTGAAGAGAATCAATAGTCGACgaaaactcctccttaagat | Anneal with oFS42 to assemble BM7 dsDNA oligomer. |
| oFS83 | aacttgctatttctagctctaaaacTTTTTTATCGGAGCTCCGGAGTCGACgaaaactcctccttaagat | Anneal with oFS43 to assemble BM8 dsDNA oligomer. |
| oFS84 | aacttgctatttctagctctaaaacCAGTGATAGCCTGATCTTCAGTCGACgaaaactcctccttaagat | Anneal with oFS44 to assemble BM9 dsDNA oligomer. |
| oFS85 | aacttgctatttctagctctaaaacATTTCGTAAACGGTATCGGTGTCGACgaaaactcctccttaagat | Anneal with oFS45 to assemble BM10 dsDNA oligomer. |
| oFS86 | aacttgctatttctagctctaaaacGTGACAGTAATATCAGAAATGTCGACgaaaactcctccttaagat | Anneal with oFS46 to assemble BM11 dsDNA oligomer. |
| oFS87 | aacttgctatttctagctctaaaacTCCGTACAACCCACAACCCTGTCGACgaaaactcctccttaagat | Anneal with oFS47 to assemble BM12 dsDNA oligomer. |
| oFS105 | gagcttatgcaattcaagtaggtactgcaaac | Screening and sequencing of *pyrE* genomic locus. |
| oFS106 | catcaaagctatactattttccgtatttacatttggg | Screening and sequencing of *pyrE* genomic locus. |
| oFS109 | caattgttcaaaaaaataatggcggcgcgccCCTGTAATCGGAGCATCTGG | Amplify *pyrE* LHA with oFS119 to assemble the *pyrE* knock-out vector. |
| oFS112 | catttgcaggcttcttatttttatgcgatcgcGTACCGCTGCTATCTGCC | Amplify *pyrE* RHA with oFS120 to assemble the *pyrE* knock-out vector. |
| oFS119 | ATCCATAACTGTCCTCCTAAATTATTCCTC | Amplify *pyrE* LHA with oFS109 to assemble the *pyrE* knock-out vector. |
| oFS120 | AAATAAGTCGAAAAAATCAATGCACGATGC | Amplify *pyrE* RHA with oFS112 to assemble the *pyrE* knock-out vector. |
| oFS57 | GAAACTTAATCATATGCGCTAAGG | Sequencing of Cas9. |
| oFS58 | ATGGATAAGAAATACTCAATAGGCTTAG | Sequencing of Cas9. |
| oFS59 | GCTTTGTCATTGGGTTTGAC | Sequencing of Cas9. |
| oFS60 | GTCGATAAAGGTGCTTCAGC | Sequencing of Cas9. |
| oFS61 | GAACATATTGCAAATTTAGCTGG | Sequencing of Cas9. |
| oFS62 | CTGACTTCCGAAAAGATTTCC | Sequencing of Cas9. |
| oFS63 | GAGTTAGAAAACGGTCGTAAACG | Sequencing of Cas9. |
| oFS68 | GCAAAATACATTCGTTGATG | Sequencing of pMTL vector series/confirm plasmid loss. |
| oFS69 | GTCAAGTATGAAATCATAAATAAAG | Sequencing of pMTL vector series. |
| oFS70 | GATAAATAGTTAACTTCAGGTTTGTC | Sequencing of pMTL vector series. |
| oFS71 | CTGTGGATAACCGTATTACC | Sequencing of pMTL vector series. |
| oFS72 | CAAGAAGAGCGACTTCGC | Sequencing of pMTL vector series. |
| oFS73 | CTAGATTTATATTTAGTCCCTTGCCTTGC | Sequencing of pMTL vector series. |
| oFS74 | CTGTTATGCCTTTTGACTATC | Sequencing of pMTL vector series. |
| oFS75 | GTCAAAATACTCTTTTCTGTTCC | Sequencing of pMTL vector series/confirm plasmid loss. |
| oFS77 | CATTGAAAGAAGTAGGAGCAC | Sequencing of *pyrE* complementation homologous region. |
| oFS88 | GGTCATAGCTGTTTCCTG | Sequencing/cPCR of pMTL vector series. |
| oFS105 | gagcttatgcaattcaagtaggtactgcaaac | Screen *pyrE* for bookmark complementation. |
| oFS106 | catcaaagctatactattttccgtatttacatttggg | Screen *pyrE* for bookmark complementation. |
| oFS208 | TGCATAGTAGACAGAAGAGC | Sequencing of *pyrE* complementation homologous region. |
| oFS215 | AATCAATGCACGATGCAG | Sequencing of *pyrE* complementation homologous region. |

The following dsDNA was ordered from IDT DNA technology:

**Table S3.** DNA synthesis summary.

| **Name** | **Sequence** | **Usage** |
| --- | --- | --- |
| **BMa** | GCATCGTGCATTGATTTTTTCGACTTATTTAGGGTTGTGGGTTGTACGGAAGGATTTCTGATATTACTGTCACAGGACCGATACCGTTTACGAAATAGGTGAAGATCAGGCTATCACTGAGGTCCGGAGCTCCGATAAAAAATGGTATTGATTCTCTTCAAGTAGAGGCCATTGTACTATCATGCTAGAGGATGCAGTCGGCTGTAGAAAGAGGCGACTGCATTTTATTATGTAAGGATCCATAACTGTCCTCCTAAATTATTCCTC | HiFi with amplicon of oFS109-oFS119 and oFS112-oFS120, and with pMTL431511-CLAU-pyrE digested with AscI and AsisI to make *pyrE* knock-out vector. |

## sgRNA design

All the seed sequences used to design sgRNAs to target protospacers in this study were either picked from literature or designed using the Benchling sgRNA design tool (<https://benchling.com>, 2017).

**Table S4.** Seed sequences used in sgRNA. For each seed sequence, the original publication and the vector in which it was assembled are given.

| Seed | Sequence | Publication | Construct |
| --- | --- | --- | --- |
| BM4 | AGGGTTGTGGGTTGTACGGA | Jiang et al., 2013 | pMTL431511_BM4 |
| BM5 | ATTTCTGATATTACTGTCAC | Jiang et al., 2013 | pMTL431511_BM5 |
| BM6 | ACCGATACCGTTTACGAAAT | Jiang et al., 2013 | pMTL431511_BM6 |
| BM7 | TGAAGATCAGGCTATCACTG | Altenbuchner, 2016 | pMTL431511_BM7 |
| BM8 | TCCGGAGCTCCGATAAAAAA | Altenbuchner, 2016 | pMTL431511_BM8 |
| BM9 | TATTGATTCTCTTCAAGTAG | Altenbuchner, 2016 | pMTL431511_BM9 |
| BM10 | CCATTGTACTATCATGCTAG | Oh & Van Pijkeren, 2014 | pMTL431511_BM10 |
| BM11 | ATGCAGTCGGCTGTAGAAAG | Oh & Van Pijkeren, 2014 | pMTL431511_BM11 |
| BM12 | CGACTGCATTTTATTATGTA | Oh & Van Pijkeren, 2014 | pMTL431511_BM12 |
| pyrE | CTATGAACTTGCAAGGCAAA | Ingle et al., 2019 | pMTL431511_BMa |

## Complementation efficiency calculation

**Table S5.** Raw data from the calculation of the complementation efficiency of each bookmark protospacer, across three independent conjugations.

| Bookmark protospacer | Replicate | | Colony count (CFU) | Complemented | Screened | Efficiency |
| --- | --- | --- | --- | --- | --- | --- |
| BM4 | I | 1132 | | 6 | 8 | 75% |
| BM4 | II | 750 | | 8 | 8 | 100% |
| BM4 | III | 1154 | | 9 | 9 | 100% |
| BM5 | I | 1616 | | 8 | 9 | 89% |
| BM5 | II | 1475 | | 7 | 7 | 100% |
| BM5 | III | 536 | | 11 | 11 | 100% |
| BM6 | I | 414 | | 12 | 12 | 100% |
| BM6 | II | 946 | | 6 | 7 | 86% |
| BM6 | III | 113 | | 7 | 7 | 100% |
| BM7 | I | 849 | | 3 | 5 | 60% |
| BM7 | II | 615 | | 3 | 3 | 100% |
| BM7 | III | 971 | | 8 | 9 | 89% |
| BM8 | I | 1178 | | 3 | 3 | 100% |
| BM8 | II | 308 | | 8 | 9 | 89% |
| BM8 | III | 433 | | 4 | 4 | 100% |
| BM9 | I | 412 | | 7 | 7 | 100% |
| BM9 | II | 690 | | 7 | 7 | 100% |
| BM9 | III | 481 | | 5 | 5 | 100% |
| BM10 | I | 410 | | 5 | 6 | 83% |
| BM10 | II | 308 | | 2 | 2 | 100% |
| BM10 | III | 436 | | 6 | 7 | 86% |
| BM11 | I | 183 | | 5 | 5 | 100% |
| BM11 | II | 156 | | 7 | 8 | 88% |
| BM11 | III | 259 | | 9 | 11 | 82% |
| BM12 | I | 235 | | 2 | 6 | 33% |
| BM12 | II | 280 | | 4 | 4 | 100% |
| BM12 | III | 568 | | 4 | 4 | 100% |
| WT | I | 0 | | N/A | N/A | N/A |
| WT | II | 0 | | N/A | N/A | N/A |
| WT | III | 0 | | N/A | N/A | N/A |

## Calculation of the off-target scores of the bookmark protospacers

The calculation of the off-target efficiency (specificity score) were done with Benchling and are detailed in Table S.5 with the parameters of the algorithm.

**Table S2.** Off-target efficiency (specificity score) of all 9 bookmark protospacers calculated with Benchling with the algorithm of Hsu et al, 2013 [1]. A score above 50 is generally considered a suitable protospacer.

| Reference genome: | | GCA_000484505.1 (*Clostridium autoethanogenum* DSM 10061) | | |
| --- | --- | --- | --- | --- |
| Design type | | Single guide | | |
| Guide length | | 20bp | | |
| PAM | | NGG (SpCas9, 3'side) | | |
| Bookmark protospacer | **Sequence** | | **PAM** | **Specificity Score** |
| BM4 | AGGGTTGTGGGTTGTACGGA | | AGG | 100 |
| BM5 | ATTTCTGATATTACTGTCAC | | AGG | 99.95 |
| BM6 | ACCGATACCGTTTACGAAAT | | AGG | 99.8 |
| BM7 | TGAAGATCAGGCTATCACTG | | AGG | 98.2 |
| BM8 | TCCGGAGCTCCGATAAAAAA | | TGG | 100 |
| BM9 | TATTGATTCTCTTCAAGTAG | | AGG | 99.5 |
| BM10 | CCATTGTACTATCATGCTAG | | AGG | 100 |
| BM11 | ATGCAGTCGGCTGTAGAAAG | | AGG | 100 |
| BM12 | CGACTGCATTTTATTATGTA | | AGG | 100 |

## Uncropped gel

**Figure S.1.** Uncropped electrophoresis gel of 6 *Clostridium autoethanogenum* colonies obtained after conjugation of pMTL431511_BMa. A 20kb band present in all lanes was dismissed from the analysis of the gel, and removed from the main text to improve clarity. Indeed, this band appears not only in the negative control ( - ) where no DNA template was present, it also appears in the empty lanes of the gel, where no PCR reaction had been loaded. We assume this band must have been the result of some kind of DNA contamination of the TAE buffer.


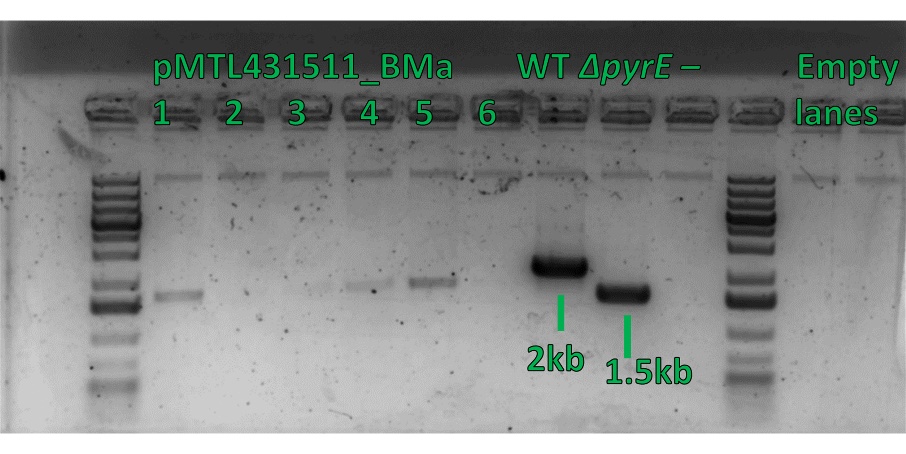


**Figure S.2.** Subsequent PCRs of the *ΔpyrE::BMa* strain screened in Figure S1. In accordance with the hypothesis that the 20 kb band observed in Figure S.1 could be safely dismissed as the product of a contamination of the TAE buffer, subsequent PCRs of the strains used as template in Figure S.1 did not exhibit any 20 kb band.


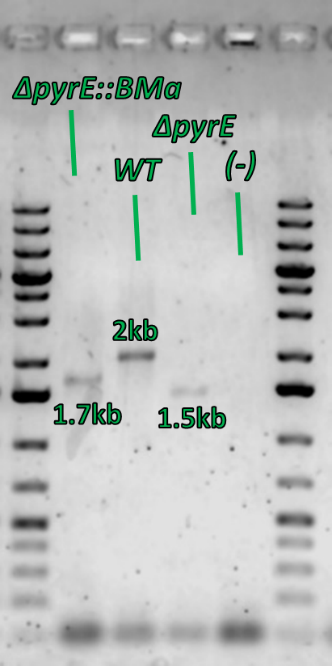


## Complementation efficiency gels

For each gel, a red line has been drawn in between the 2 kb bands of the DNA ladder. The strains complemented to the WT genotype should give a 2 kb amplicon while the Δ*pyrE*::*BM4-12* strain should give a 1.7 kb amplicon. The different replicates are separated by green vertical lines and labelled with roman numerals (I for first replicate, II for second replicate and III for third replicate). Many colonies gave no amplicons at all, which is a limitation of our colony PCR protocol. These colonies were excluded from the calculation of the complementation efficiency.

### pMTL83151_BM4

**Figure S.3.** Electrophoresis gel of *Clostridium autoethanogenum ΔpyrE::BM4-12* colonies obtained after conjugation of pMTL431511_BM4. The *pyrE* locus of each colony was amplified using the primers oFS105 and oFS106 then run on a 1% or 2% (w/v) agarose gel. The expected size of the amplicon of a successfully complemented *pyrE* locus is 2 kb, versus 1.7 kb for the *ΔpyrE::BM4-12* background. The different replicates are separated by green vertical lines and labelled with roman numerals. I = first replicate; II = second replicate; III = third replicate; WT= wild-type *pyrE* locus (2 kb); (-) = negative control of colony PCR without DNA template; Col.: colony.


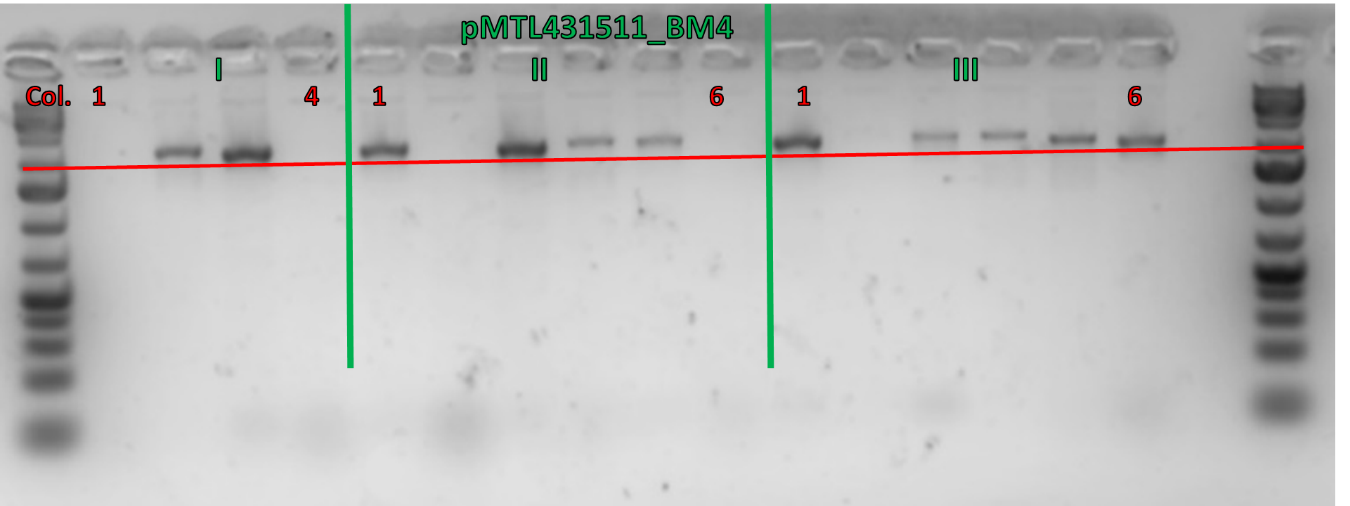


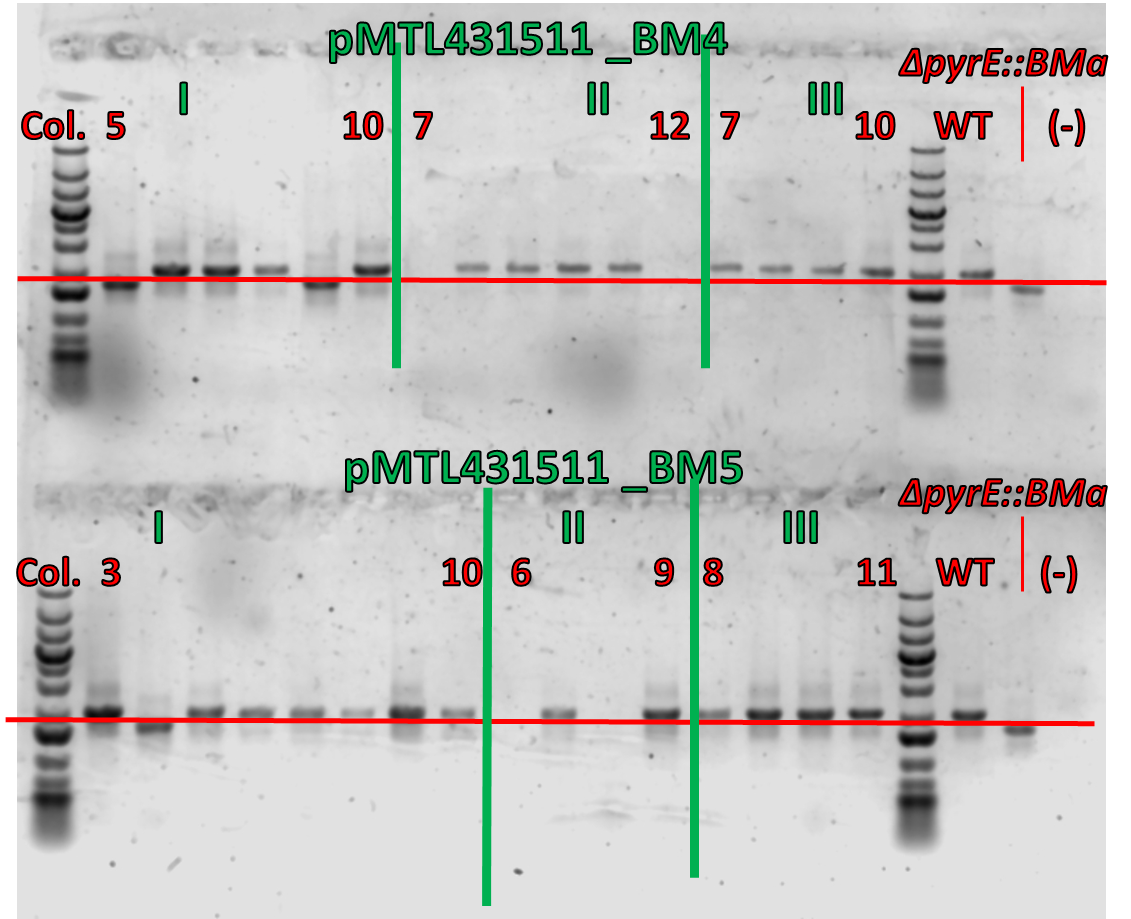


### pMTL83151_BM5

**Figure S.4.** Electrophoresis gel of *Clostridium autoethanogenum ΔpyrE::BM4-12* colonies obtained after conjugation of pMTL431511_BM5. The *pyrE* locus of each colony was amplified using the primers oFS105 and oFS106 then run on a 1% or 2% (w/v) agarose gel. The expected size of the amplicon of a successfully complemented *pyrE* locus is 2 kb, versus 1.7 kb for the *ΔpyrE::BM4-12* background. The different replicates are separated by green vertical lines and labelled with roman numerals. I = first replicate; II = second replicate; III = third replicate; WT= wild-type *pyrE* locus (2 kb); (-) = negative control of colony PCR without DNA template; Col.: colony.


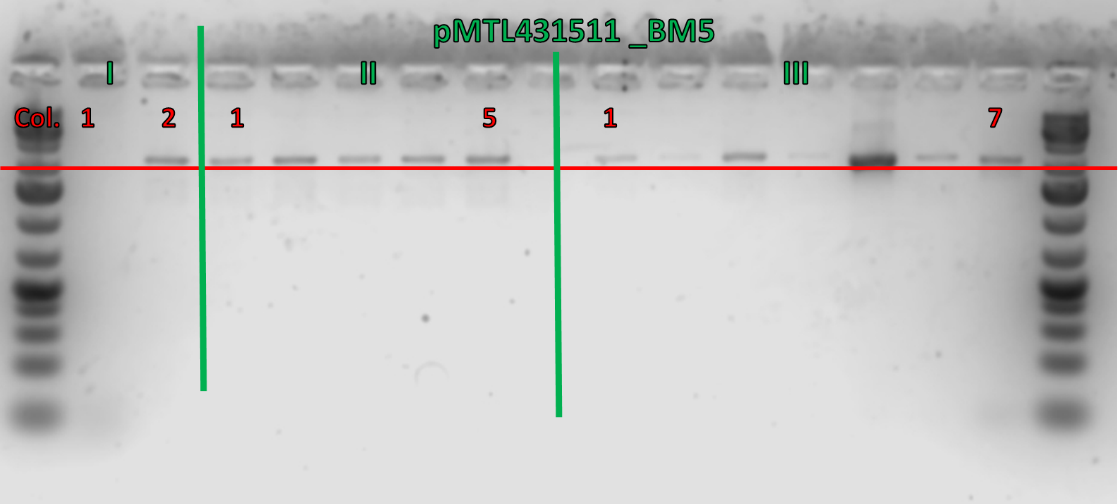


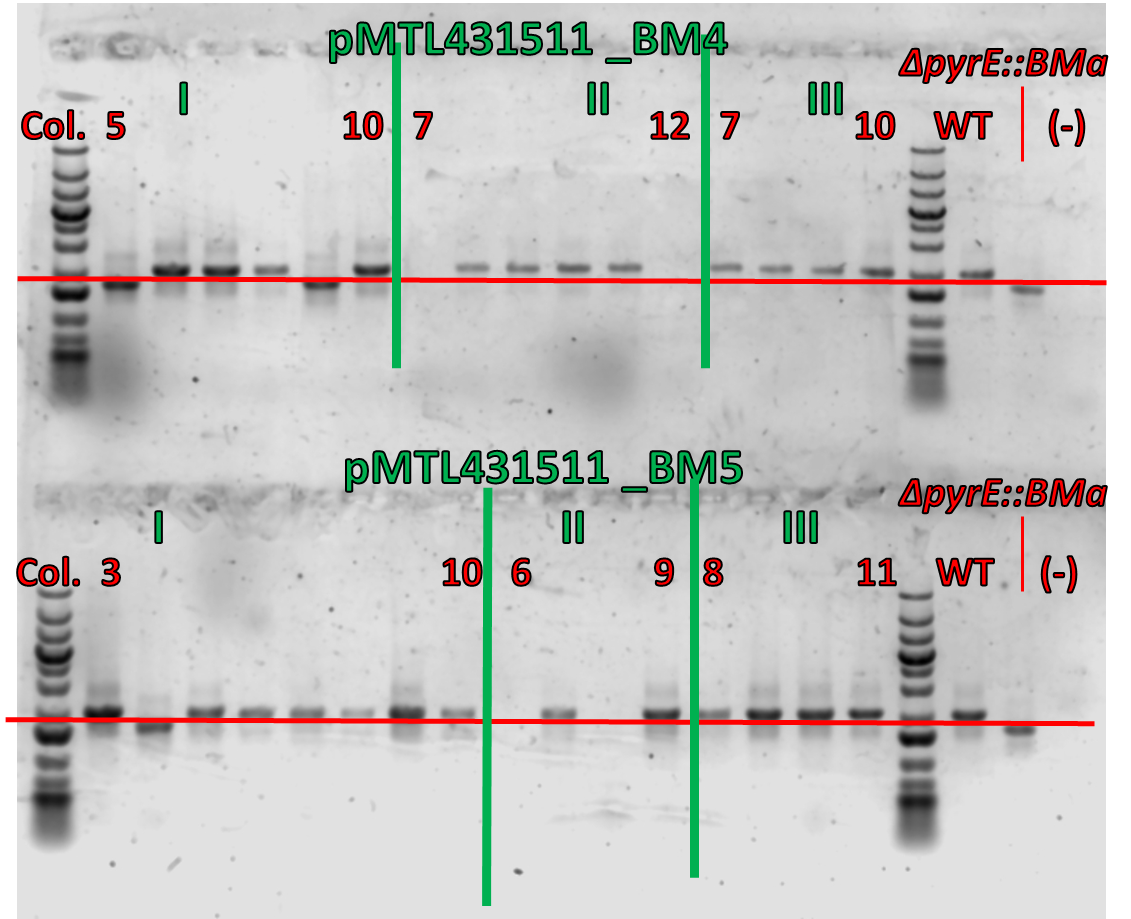


### pMTL83151_BM6

**Figure S.5.** Electrophoresis gel of *Clostridium autoethanogenum ΔpyrE::BM4-12* colonies obtained after conjugation of pMTL431511_BM6. The *pyrE* locus of each colony was amplified using the primers oFS105 and oFS106 then run on a 1% or 2% (w/v) agarose gel. The expected size of the amplicon of a successfully complemented *pyrE* locus is 2 kb, versus 1.7 kb for the *ΔpyrE::BM4-12* background. The different replicates are separated by green vertical lines and labelled with roman numerals. I = first replicate; II = second replicate; III = third replicate; WT= wild-type *pyrE* locus (2kb); (-) = negative control of colony PCR without DNA template; Col.: colony.


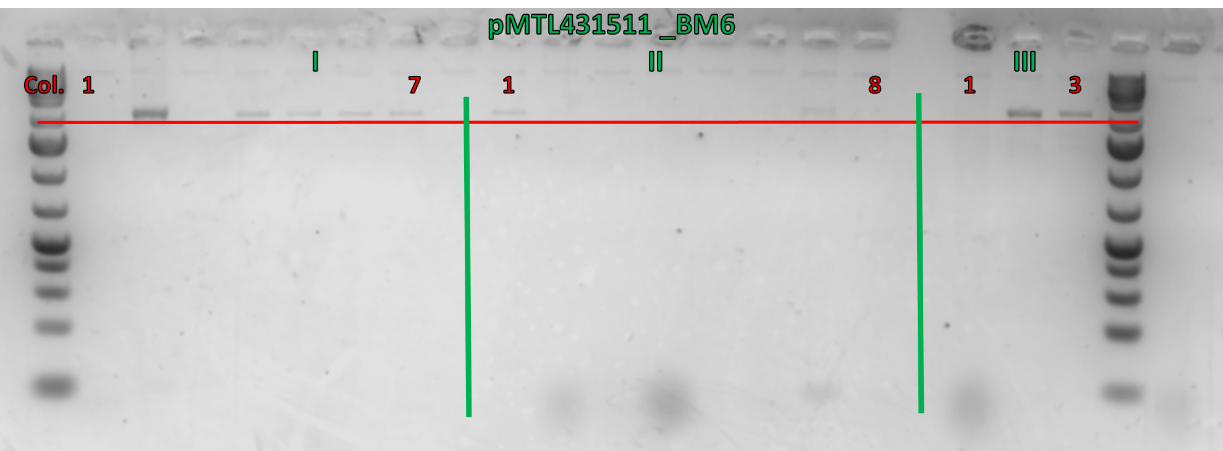


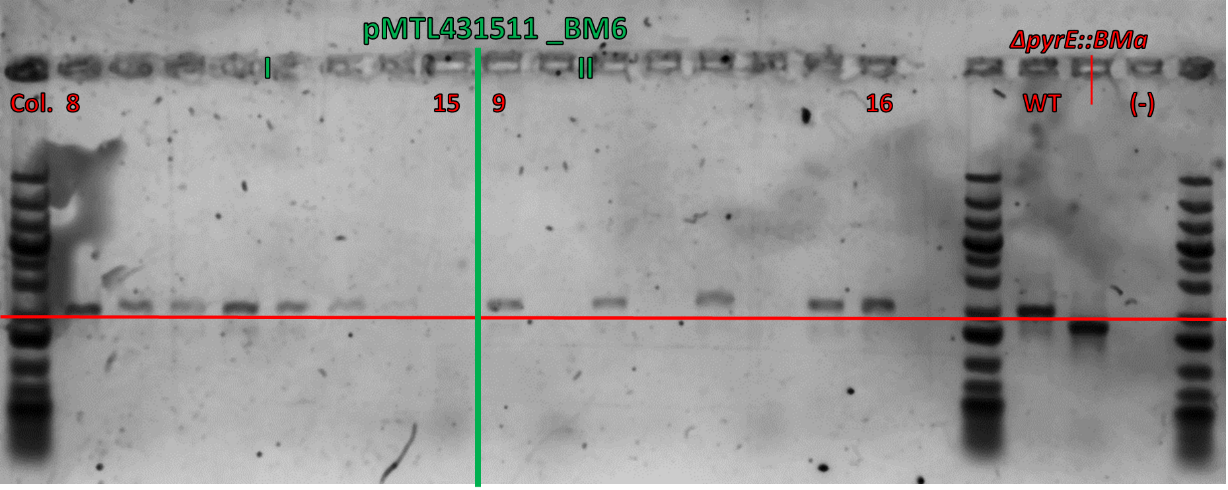


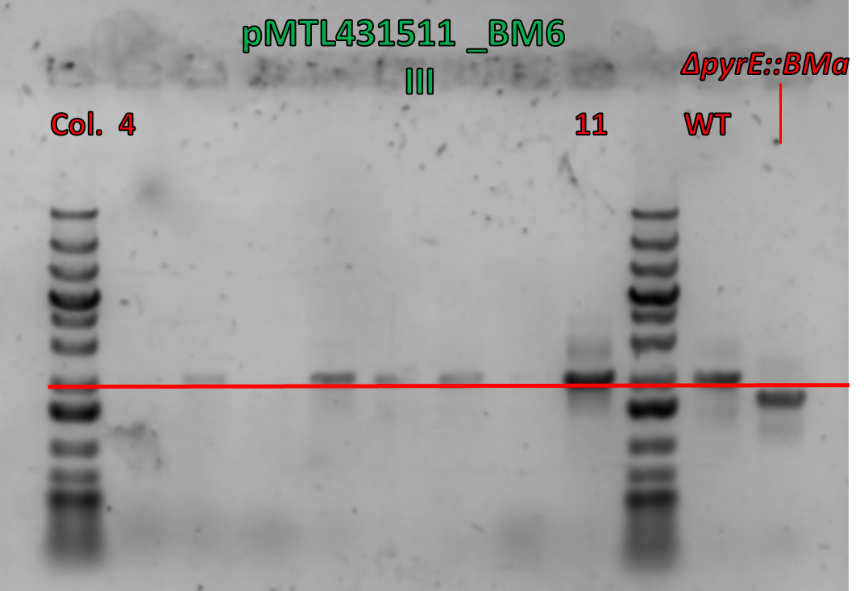


### pMTL83151_BM7

**Figure S.6.** Electrophoresis gel of *Clostridium autoethanogenum ΔpyrE::BM4-12* colonies obtained after conjugation of pMTL431511_BM7. The *pyrE* locus of each colony was amplified using the primers oFS105 and oFS106 then run on a 1% (w/v) agarose gel. The expected size of the amplicon of a successfully complemented *pyrE* locus is 2 kb, versus 1.7 kb for the *ΔpyrE::BM4-12* background. The different replicates are separated by green vertical lines and labelled with roman numerals. I = first replicate; II = second replicate; III = third replicate; WT= wild-type *pyrE* locus (2kb); (-) = negative control of colony PCR without DNA template; Col.: colony.


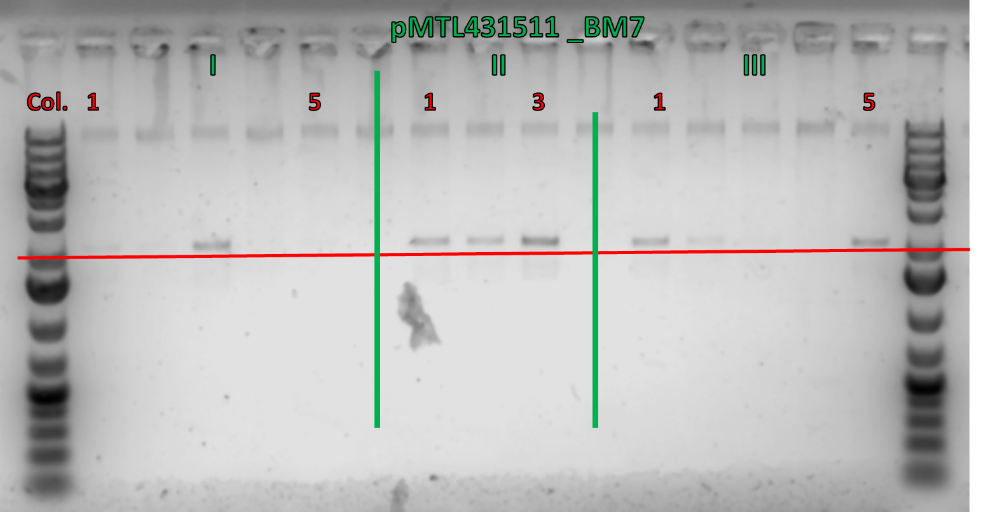


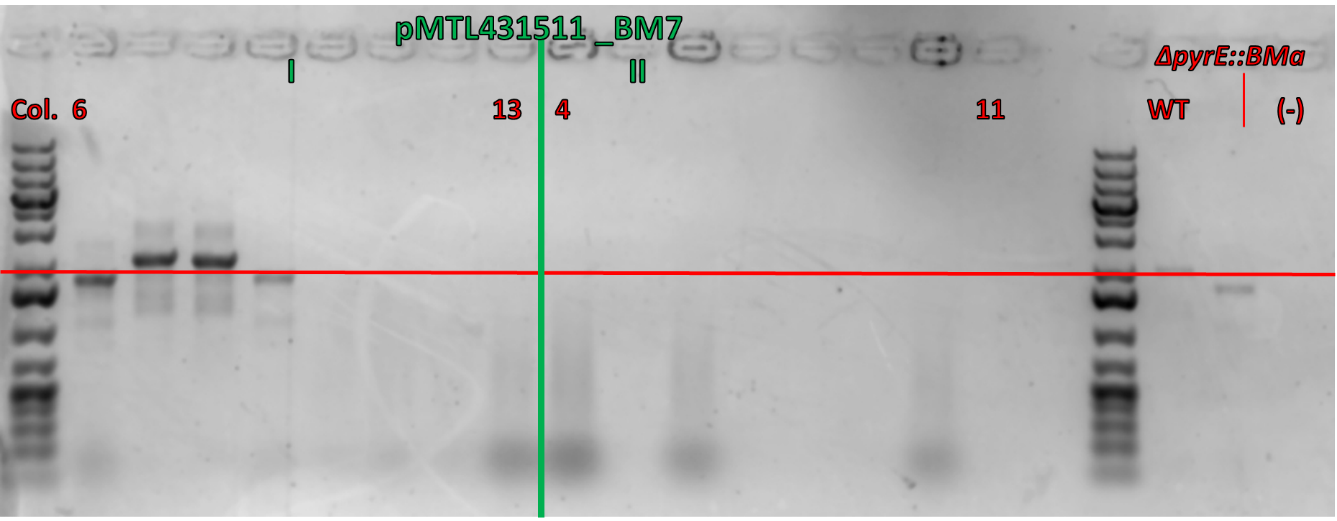


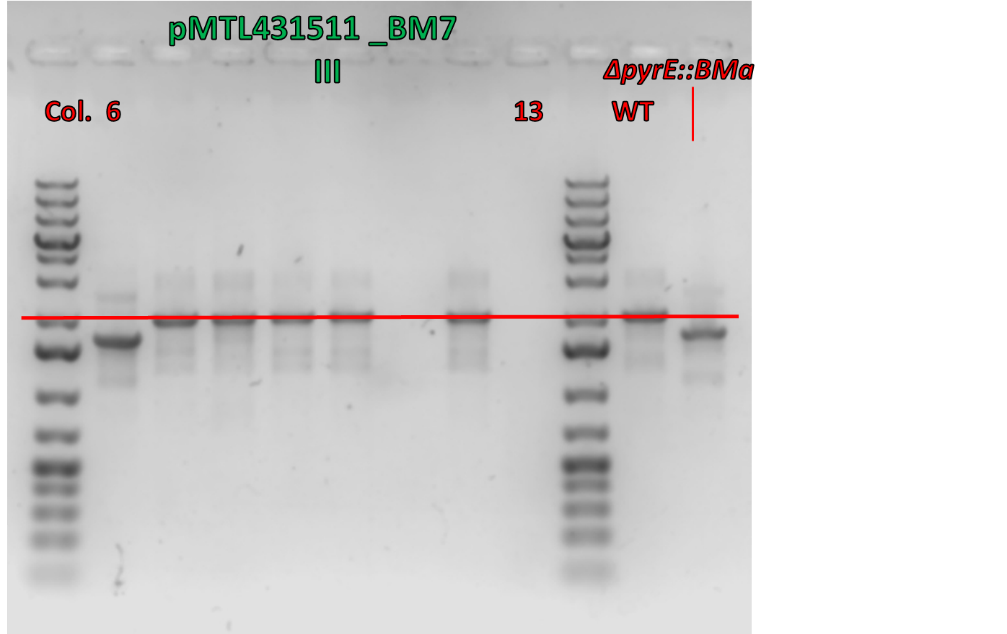


### pMTL83151_BM8

**Figure S.7.** Electrophoresis gel of *Clostridium autoethanogenum ΔpyrE::BM4-12* colonies obtained after conjugation of pMTL431511_BM8. The *pyrE* locus of each colony was amplified using the primers oFS105 and oFS106 then run on a 1% (w/v) agarose gel. The expected size of the amplicon of a successfully complemented *pyrE* locus is 2 kb, versus 1.7 kb for the *ΔpyrE::BM4-12* background. The different replicates are separated by green vertical lines and labelled with roman numerals. I = first replicate; II = second replicate; III = third replicate; WT= wild-type *pyrE* locus (2 kb); (-) = negative control of colony PCR without DNA template; Col.: colony.


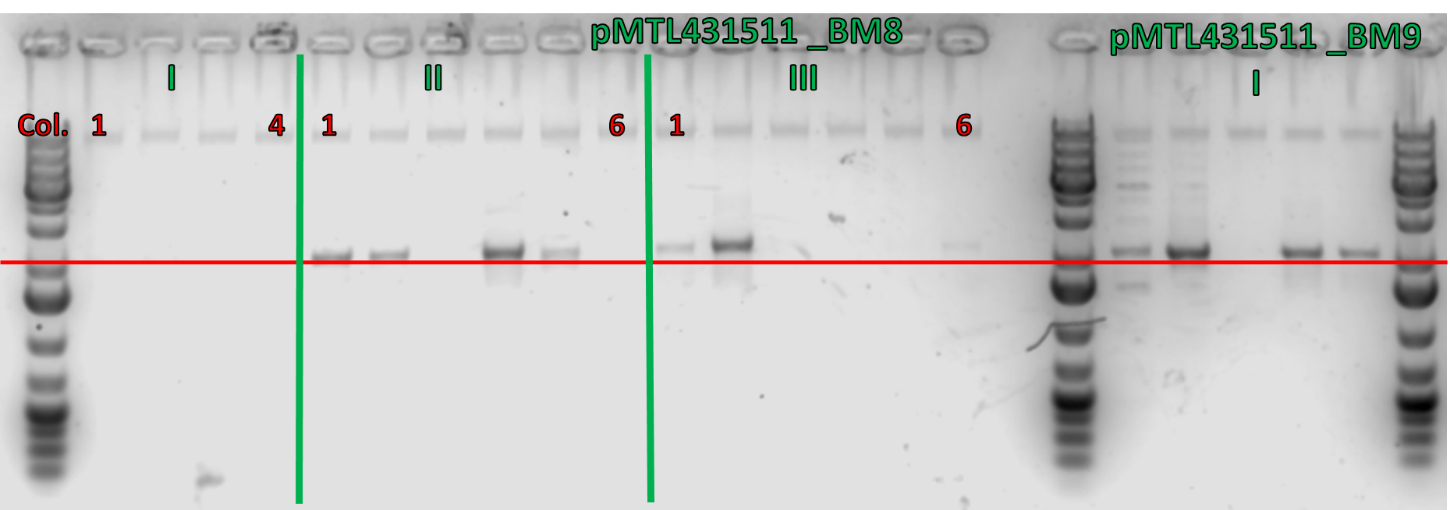


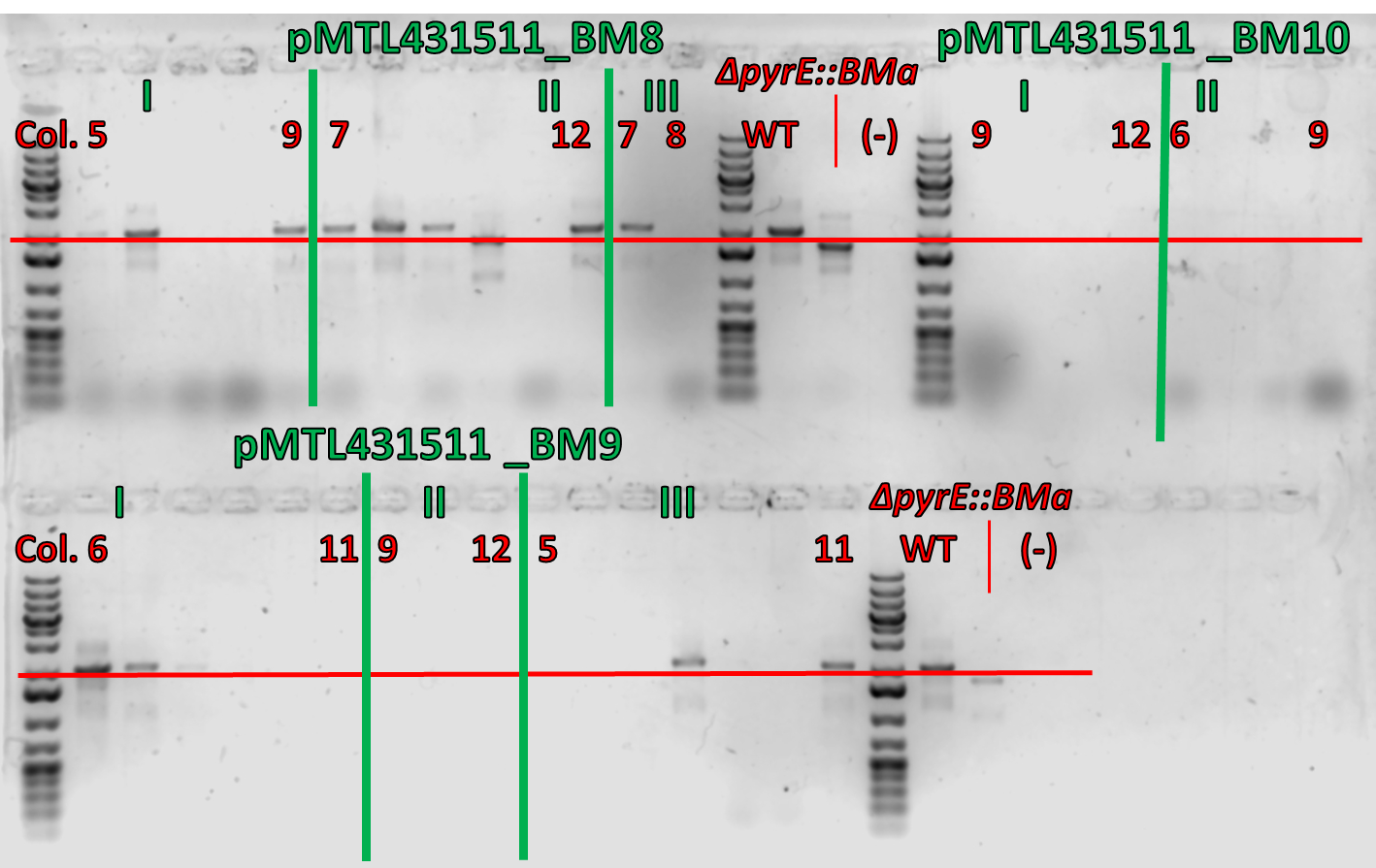


### pMTL83151_BM9

**Figure S.8.** Electrophoresis gel of *Clostridium autoethanogenum ΔpyrE::BM4-12* colonies obtained after conjugation of pMTL431511_BM9. The *pyrE* locus of each colony was amplified using the primers oFS105 and oFS106 then run on a 1% (w/v) agarose gel. The expected size of the amplicon of a successfully complemented *pyrE* locus is 2 kb, versus 1.7 kb for the *ΔpyrE::BM4-12* background. The different replicates are separated by green vertical lines and labelled with roman numerals. I = first replicate; II = second replicate; III = third replicate; WT= wild-type *pyrE* locus (2 kb); (-) = negative control of colony PCR without DNA template; Col.: colony.


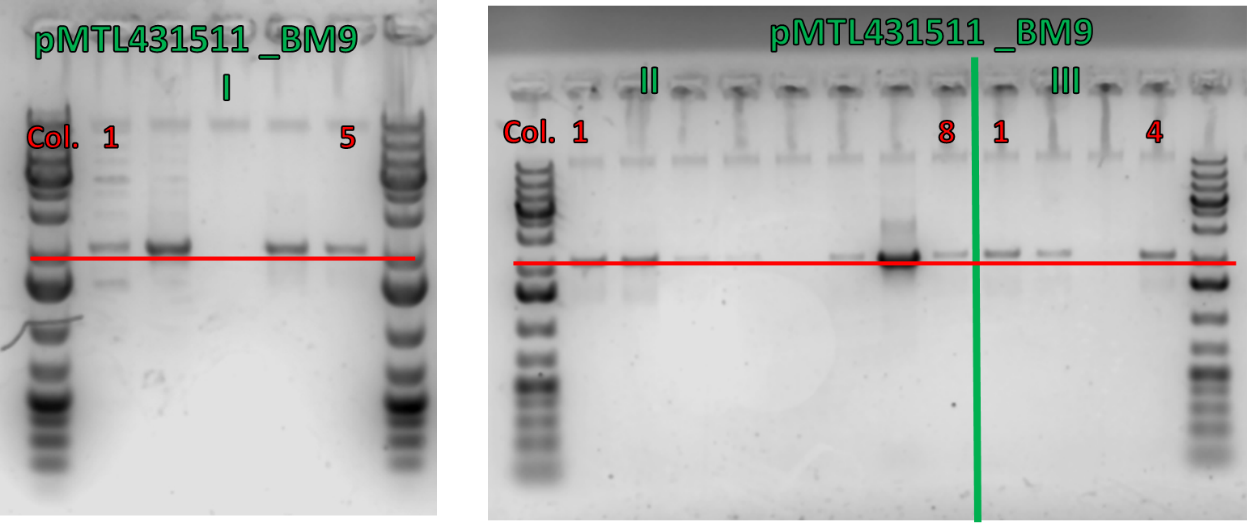


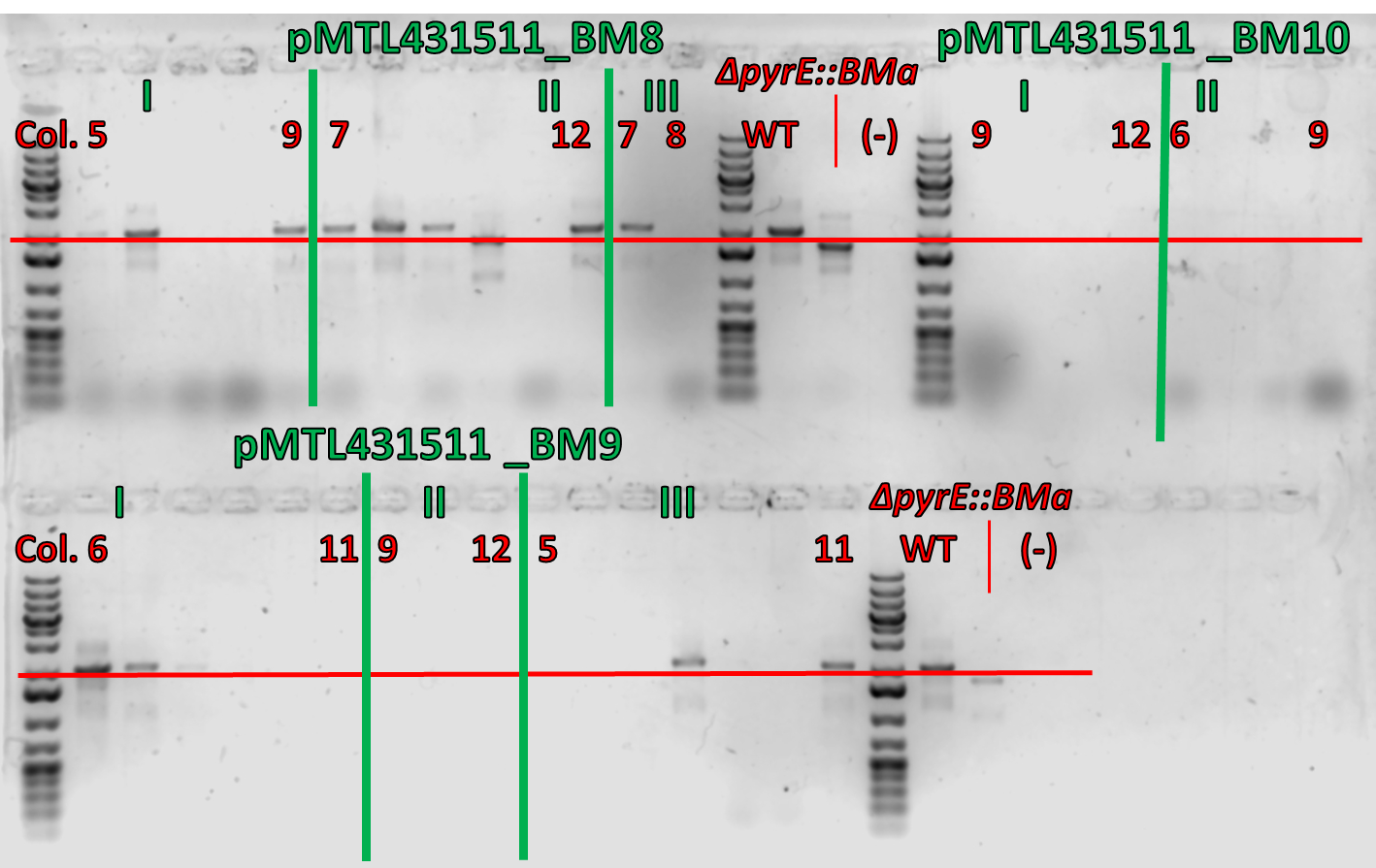


### pMTL83151_BM10

**Figure S.9.** Electrophoresis gel of *Clostridium autoethanogenum ΔpyrE::BM4-12* colonies obtained after conjugation of pMTL431511_BM10. The *pyrE* locus of each colony was amplified using the primers oFS105 and oFS106 then run on a 1% (w/v) agarose gel. The expected size of the amplicon of a successfully complemented *pyrE* locus is 2 kb, versus 1.7 kb for the *ΔpyrE::BM4-12* background. The different replicates are separated by green vertical lines and labelled with roman numerals. I = first replicate; II = second replicate; III = third replicate; WT= wild-type *pyrE* locus (2 kb); (-) = negative control of colony PCR without DNA template; Col.: colony.


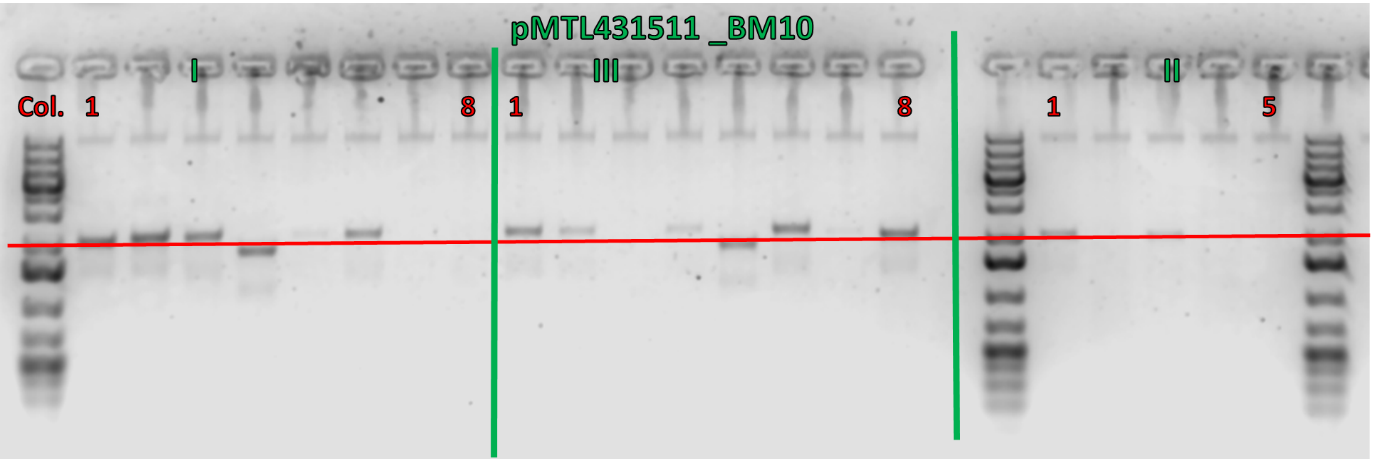


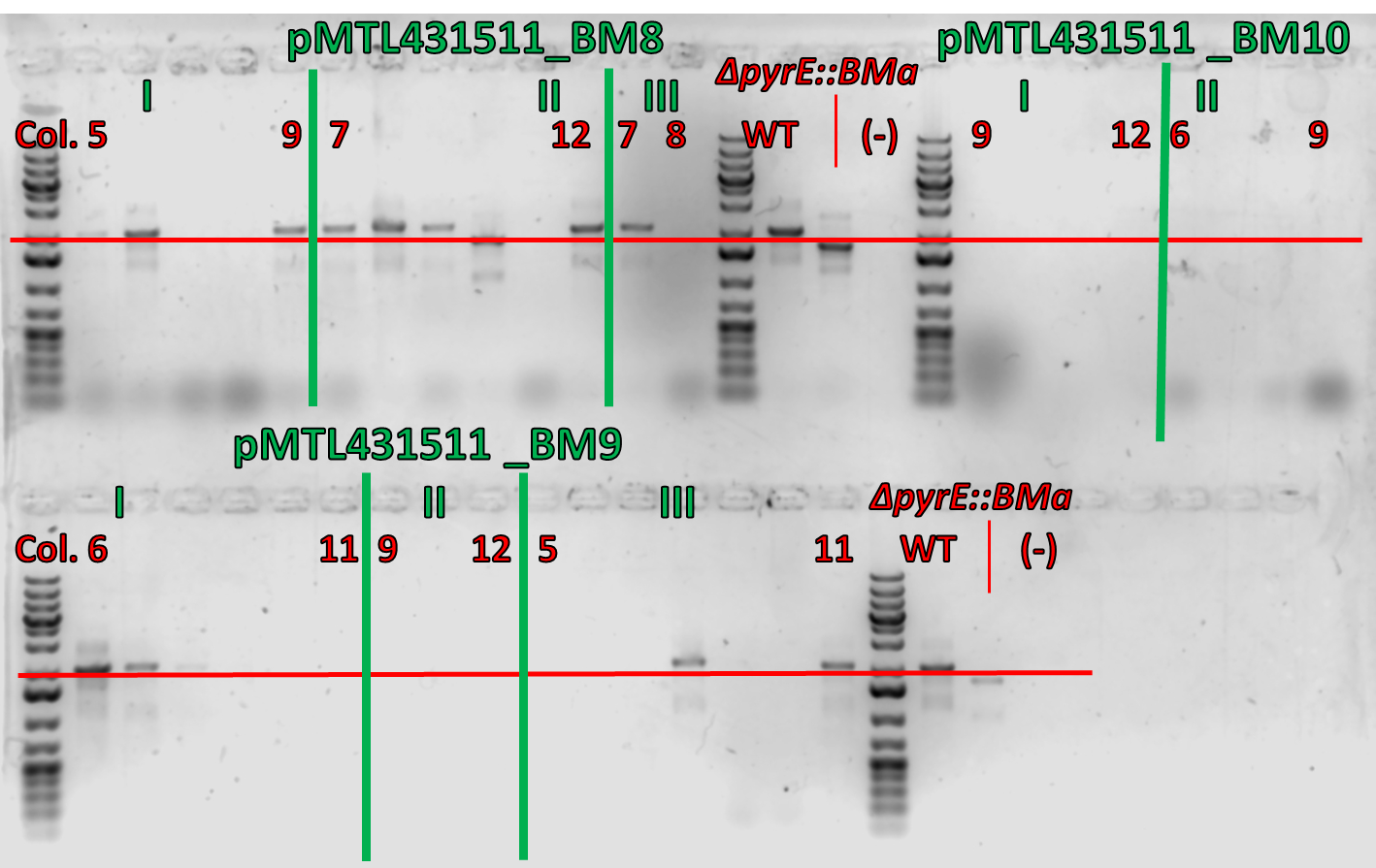

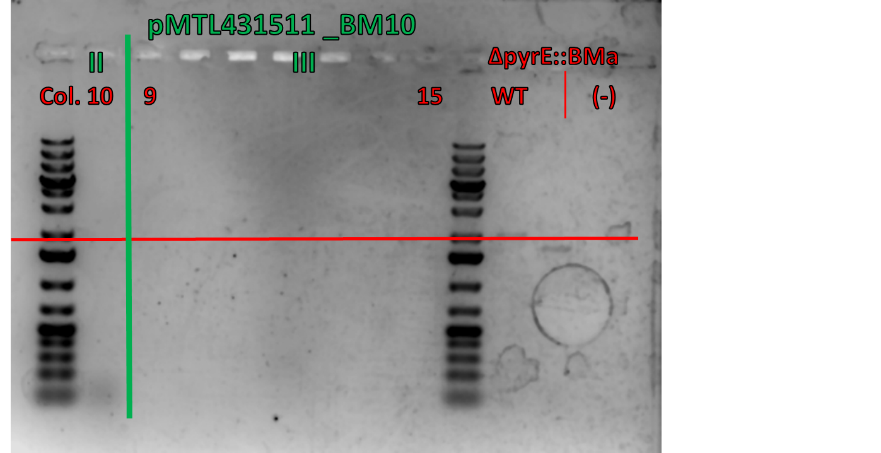


### pMTL83151_BM11

**Figure S.10.** Electrophoresis gel of *Clostridium autoethanogenum ΔpyrE::BM4-12* colonies obtained after conjugation of pMTL431511_BM11. The *pyrE* locus of each colony was amplified using the primers oFS105 and oFS106 then run on a 1% (w/v) agarose gel. The expected size of the amplicon of a successfully complemented *pyrE* locus is 2 kb, versus 1.7 kb for the *ΔpyrE::BM4-12* background. The different replicates are separated by green vertical lines and labelled with roman numerals. I = first replicate; II = second replicate; III = third replicate; WT= wild-type *pyrE* locus (2 kb); (-) = negative control of colony PCR without DNA template; Col.: colony.


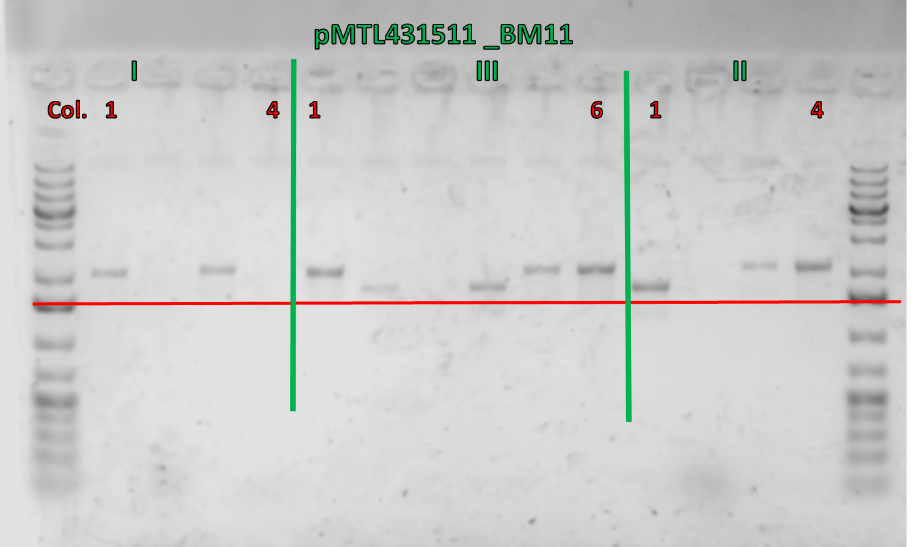


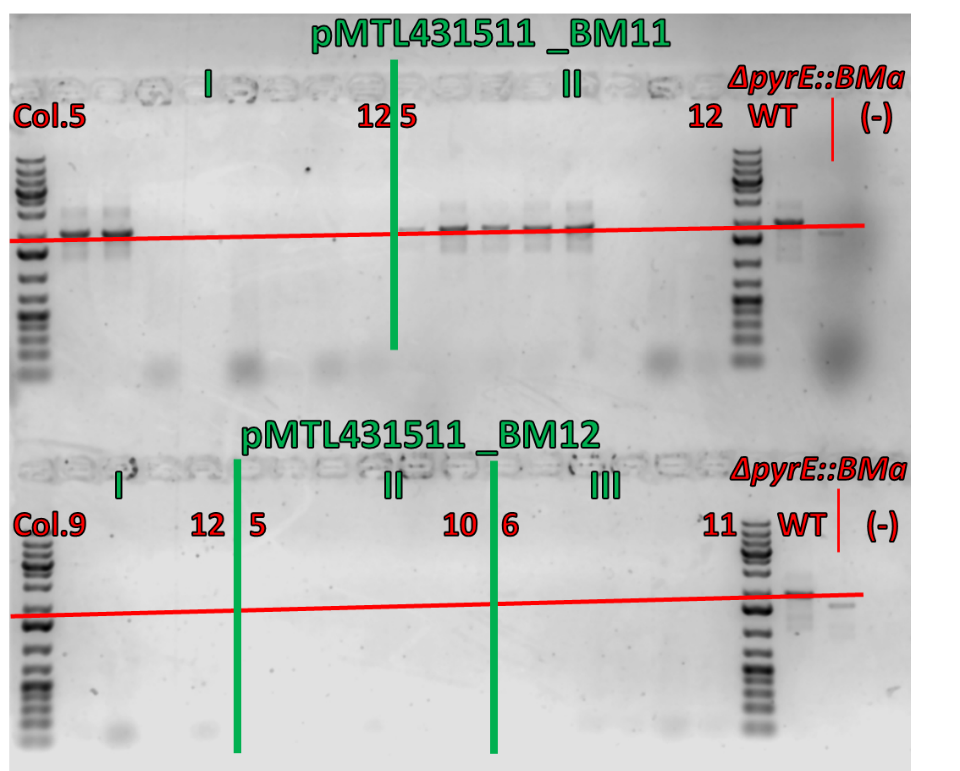


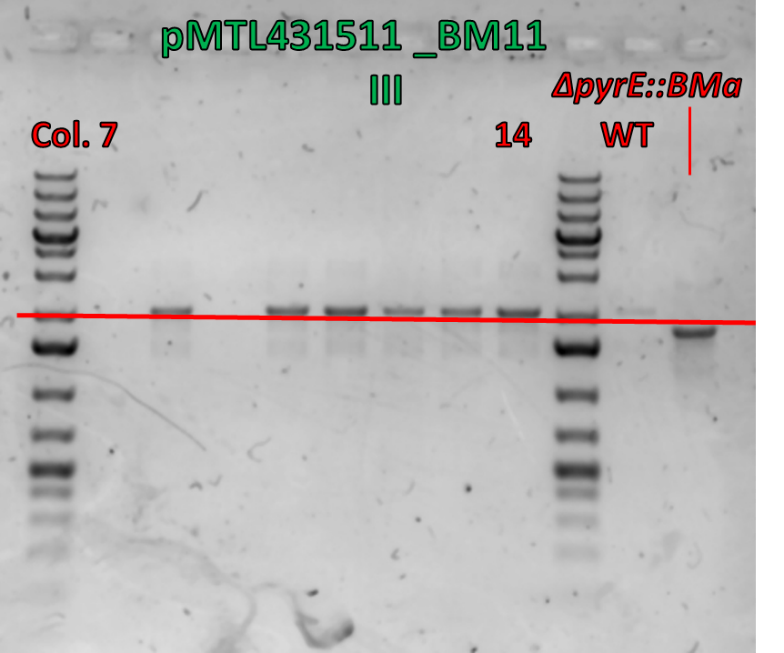


### pMTL83151_BM12

**Figure S.11.** Electrophoresis gel of *Clostridium autoethanogenum ΔpyrE::BM4-12* colonies obtained after conjugation of pMTL431511_BM12. The *pyrE* locus of each colony was amplified using the primers oFS105 and oFS106 then run on a 1% (w/v) agarose gel. The expected size of the amplicon of a successfully complemented *pyrE* locus is 2 kb, versus 1.7 kb for the *ΔpyrE::BM4-12* background. The different replicates are separated by green vertical lines and labelled with roman numerals. I = first replicate; II = second replicate; III = third replicate; WT= wild-type *pyrE* locus (2 kb); (-) = negative control of colony PCR without DNA template; Col.: colony.


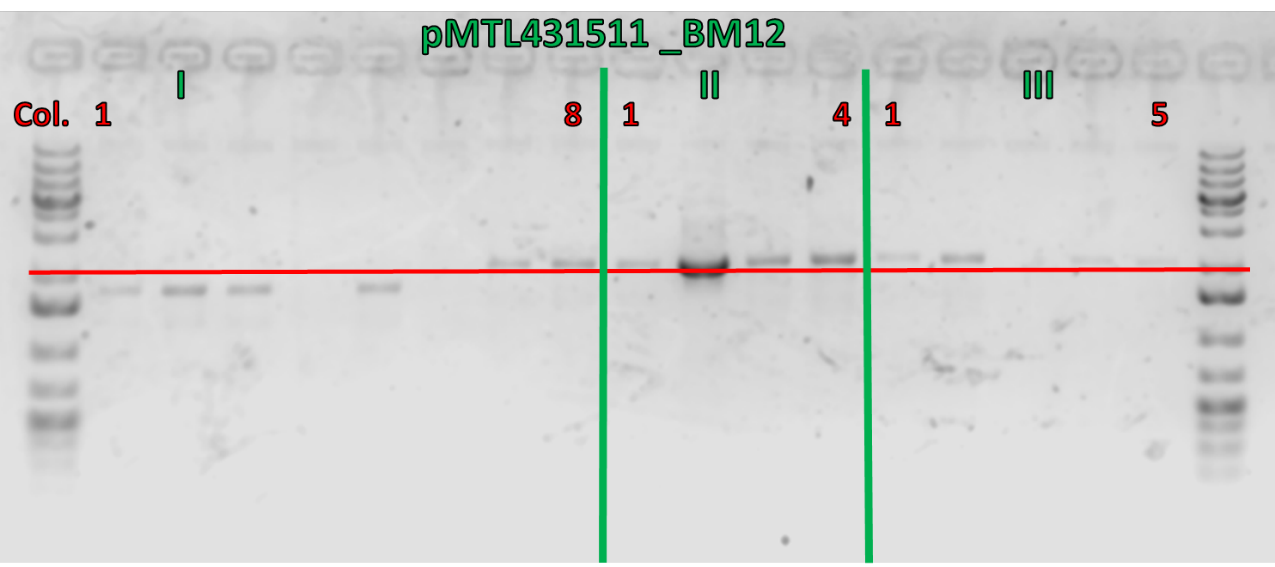


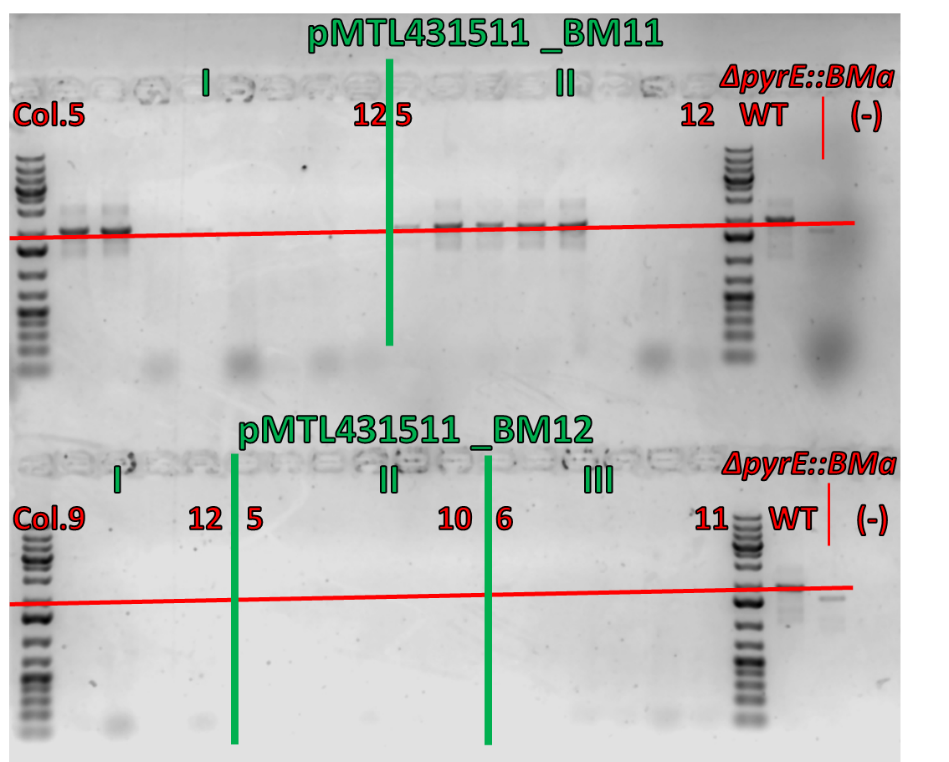


## References

1. Hsu, P.D.; Scott, D.A.; Weinstein, J.A.; Ran, F.A.; Konermann, S.; Agarwala, V.; Li, Y.; Fine, E.J.; Wu, X.; Shalem, O.; et al. DNA targeting specificity of RNA-guided Cas9 nucleases. *Nat. Biotechnol.* **2013**, *31*, 827–832.
